# Callous-Unemotional Traits Only Versus the Multidimensional Psychopathy Construct as Predictors of Various Antisocial Outcomes During Early Adolescence

**DOI:** 10.1007/s10862-018-9659-5

**Published:** 2018-03-09

**Authors:** Henrik Andershed, Olivier F. Colins, Randall T. Salekin, Alexandros Lordos, Melina Nicole Kyranides, Kostas A. Fanti

**Affiliations:** 1 School of Law, Psychology, and Social Work, Örebro University, 701 82 Örebro, SE Sweden; 2 Leiden University Medical Center, Leiden, Netherlands; 3 University of Alabama, Tuscaloosa, AL USA; 4 University of Cyprus, Nicosia, Cyprus; 5 University of Edinburgh, Department of Clinical Psychology, Edinburgh, Scotland

**Keywords:** Callous-unemotional traits, Conduct problems, Psychopathic traits, Aggression, Substance use

## Abstract

The aim of this study was to compare callous-unemotional (CU) traits versus the multidimensional psychopathy construct in their ability to predict future and stable antisocial behavior. At baseline, a community sample of 996 Cypriot 12-year old adolescents (52% girls) completed measures that tap conduct problems (CP) and psychopathic traits, including CU. CP, aggression, and substance use were self-reported at 1–3 year follow-ups. Youths were assigned to six mutually exclusive groups based on their baseline levels of CP and psychopathic traits. Youth with CP scoring high on all three psychopathic traits dimensions (*Psychopathic Personality + CP*) showed the most robust and highest risk for future and stable CP, aggression, and substance use, followed by youth who were high on all three psychopathic traits dimensions but displayed no concurrent CP (*Psychopathic Personality Only*) and CP youth with low levels of psychopathic traits (*CP Only*). Youth with CP who merely manifested callous-unemotional traits (*Callous-Unemotional + CP*) were only at risk for future CP. The findings suggest that the CU traits-based approach for subtyping children with CP is less informative compared to a subtyping approach using various psychopathic traits dimensions in predicting future and stable forms of various antisocial outcomes. These findings and their consistency with prior work indicate the need for additional research to examine the various psychopathic traits dimensions rather than focusing solely on CU traits, especially for CP subtyping purposes.

**Electronic supplementary material:**

The online version of this article (10.1007/s10862-018-9659-5) contains supplementary material, which is available to authorized users.

## Introduction

Children and adolescents with conduct problems (CP) constitute a heterogeneous group, not only in types of CP they exhibit (Lindhiem et al. 2015), but also in their risk for future antisocial outcomes (e.g., Odgers et al. 2008). Past research suggests that callous-unemotional (CU) traits help to identify a subgroup of children with CP who exhibit a more severe and stable pattern of CP compared to youth with CP low on CU traits (Frick et al. 2014). Reflecting this body of evidence, CU traits have increasingly been included in theoretical models and empirical studies on CP, and are expected to influence clinical work with children and adolescents, especially since classification systems have added (*DSM-5*), or may add (*ICD-11*) a CU-based specifier for the diagnosis of conduct disorder (APA 2013; Salekin 2016a, b, 2017). Notwithstanding the relevance of studying CU traits in relation to CP, and as detailed elsewhere in this Special Issue (see Colins, Andershed, Salekin, & Fanti 2018) it remains unclear if CU traits is the best predictor of future and stable antisocial outcomes, or if a greater representation of psychopathic traits is needed to identify the adolescents who are at the highest risk.

A recently published study among 1867 3- to 5-years old Swedish boys and girls used teacher ratings of CP and three psychopathic traits dimensions (i.e., CU, interpersonal and behavioral/lifestyle) to assign children to six mutually exclusive groups (Frogner, Gibson, Andershed, & Andershed 2016). These groups were: (1) low on CP and all three psychopathic traits dimensions (*Control*); (2) high on CP and low on all three psychopathy dimensions (*CP Only*); (3) low on CP and high on the CU traits dimension only (*Callous-Unemotional Only*); (4) low on CP and high on all three psychopathic traits dimensions (*Psychopathic Personality Only*); (5) high on CP and on the CU traits dimension only (*Callous-Unemotional + CP*); and (6) high on CP and all three psychopathic traits dimension (*Psychopathic Personality + CP*). Crucially, *Psychopathic Personality + CP* boys were at a greater risk for future and stable CP than the other groups. Overall, this finding was replicated among girls with the notable exception that *Psychopathic Personality + CP* and *Callous-Unemotional + CP* girls were equally at risk for stable CP. Colins and colleagues (2018; This special issue) tested if the findings from Frogner et al. (2016) could be replicated among 690 7- to 12-year old Cypriot boys and girls whilst using the same analytical approach but using other informants (i.e., parents) and other psychopathy measures. Results showed that *Psychopathic Personality + CP* children by far showed the most robust and highest risk for future and stable CP, whereas *CP Only* children were often equally, and sometimes even at a higher risk than *Callous-Unemotional + CP* children. Taken together, there is some evidence to suggest that using CU traits only is less sufficient than using CU traits in combination with the other psychopathic traits dimensions for identifying CP youth who are at the highest risk for future and stable CP. Prior work (Colins et al. 2018) also revealed significant prospective relations between *Psychopathic Personality Only* – and to a lesser extent also between *CU Only* – on the one hand and antisocial behavior on the other hand, suggesting that psychopathic traits that do not co-occur with baseline CP have at least some prognostic utility.

### This Study

The present study was designed to replicate and extend these aforementioned findings in various ways. First, this study will rely on self-report measures instead of teachers (Frogner et al. 2016) or parents (Colins et al., 2018, this Special Issue). Self-report measures constitute a major advancement in the assessment of psychopathic traits (e.g., Colins and Andershed 2016; Vitacco et al. 2014), and, thus, are also important to put CP subtyping approaches to the test (e.g., Colins and Andershed 2015; Kimonis et al. 2015). Second, antisocial behaviors are quite heterogeneous in kind (e.g., aggression, rule-breaking) and severity (e.g., fighting, lying, shoplifting). As such, it is relevant to know if CP subtyping approaches also help to identify youth at risk for one of the most severe forms of antisocial behavior, that is, aggression against other people. In addition, substance use has been a relevant external variable to evaluate in subtyping models (e.g., Frick et al. 2014; Wymbs et al. 2012) and can be considered an important outcome when scrutinizing the prognostic usefulness of psychopathy scores (Colins et al. 2015). Therefore, this study will also include antisocial outcomes other than CP, including aggression and substance use.

#### Hypotheses

Mirroring the expectation that children with CP who manifest psychopathic traits constitute a severe CP subgroup (e.g., Frick et al. 2014; APA 2013), it was first hypothesized that *Psychopathic Personality + CP* and *Callous-Unemotional + CP* adolescents will show the highest CP during baseline than adolescents in the other groups. Crucially, it was also expected that the *Psychopathic Personality + CP* adolescents will present the highest CP during baseline compared to *Callous-Unemotional + CP* adolescents. Second, it is expected that *Psychopathic Personality + CP* and *Callous-Unemotional + CP* adolescents will be at a higher risk for future and stable CP, aggression, and substance use, than their counterparts in the other groups. *Psychopathic Personality + CP* adolescents are nevertheless expected to be at a higher risk than *Callous-Unemotional + CP* adolescents. Finally, the present study also explored if adolescents in the *Callous-Unemotional Only* and *Psychopathic Personality Only* groups are at risk for developing the antisocial outcomes under consideration.

## Method

### Participants and Procedure

The respondents were recruited from 13 middle schools in three school districts (Nicosia, Larnaca, Limasol) in Cyprus. The sample consisted of 1274 Greek Cypriot adolescents (50.1% girls) initially, and the final sample used in the current study who completed the study’s measures across all four waves of measurement were 996 youth (52% girls; *M* age = 12.12, *SD* = .55; Boys: 479, Girls; 517). After approval of the study by the Cyprus Ministry of Education and school boards, students were given an informed consent form for their parents to sign, and only students with parental consent were permitted to participate in the study. In the classroom, students were informed about the study and about their rights as participants. Group assessments were conducted with questionnaires being administered by trained research assistants once at grade 7 and grade 8 and twice at grade 9. Attrition was due to an inability to contact students who had moved away or transferred to a different school. The sample was diverse in terms of parental educational levels (20.6% below high school education, 45.95% with a high school education, and 33.45% with a university degree) and parental marital status (7.2% came from single parent families). Parental educational level was used as a proxy for parental Socioeconomic Status (SES).

### Measures

#### Psychopathic Traits

Callous-Unemotional traits (tapping the affective psychopathic traits dimension) were measured using the self-report version of the 24-item Inventory of Callous-Unemotional Traits (ICU; Frick 2004). ICU items (e.g. “I do not feel remorseful when I do something wrong”) were rated on a 4-point Likert scale (0 = “not at all” to 3 = “definitely true”), with a higher total ICU score (α = .80) indicating a higher level of CU traits. Grandiosity traits (tapping the interpersonal psychopathic traits dimension) and Impulsive traits (tapping the behavioral/lifestyle traits dimension) were measured with the Antisocial Process Screening Device (APSD) self-report version (Frick and Hare 2001). APSD items are scored on a 3-point scale (0 = “not at all true,” 1 = “sometimes true,” and 2 = “definitely true”). For the purpose of the current investigation, the 7-item Grandiosity or Narcissism subscale (α = .73; example item: “I act charming or nice to get things I want”), and the 5-item Impulsivity subscale (α = .70; example item: “I do not plan ahead or leave things until the last moment”) of the APSD were used, based on the availability of the CU measure of the ICU in the present data. The ICU was developed to enable a more comprehensive assessment of CU traits than the APSD (e.g., Frick 2009).

**CP and Aggression** were measured with the Youth Self-Report (YSR; Achenbach 1991). Adolescents rated how well each of the items described them over the past 6 months on a 3-point scale (0 “not true” to 2 “very true or often true”). For the present study, the 15 items of the DSM-oriented conduct problem scale (α across the four waves ranged from .83 to .93), and the 17 item aggressive behavior (α across the four waves ranged from .88 to .91) subscales were used. Children were classified in the stable CP group if they presented .5 SD above mean in CP at waves 2, 3, and 4. All other children were classified in the no-stable conduct problem category. The same strategy was used to calculate stable aggression and stable substance use (see below).

**Substance Use** was measured with the Adolescent Symptom Inventory-4 (ASI-4; Gadow and Sprafkin 1998), which is a screening instrument designed to assess symptoms of several psychiatric disorders. For the current study, only the 6-item substance use subscale (α across the four waves ranged from .73 to .75; i.e., cigarettes, alcohol, and illegal drugs) was used.

### Statistical Analyses

First, Pearson product moment correlations were calculated to display the bivariate relationships among the study variables. Second, and echoing prior work in this Special Issue, a cutoff of .5 *SD* above the mean was used to dichotomize participants into high (above cut-off) and low levels (below cut-off) of baseline CP, CU traits, Grandiosity, and Impulsivity. This cut-off was chosen to enable comparison with prior work that used distribution based cut-off scores to assign children to high CU or high psychopathic traits groups (e.g., Klapwijk et al. 2015; Pasalich et al. 2012; Schwenk et al. 2012; Van Baardewijk et al. 2009; Viding et al. 2008), but also to assure that enough children were assigned to the groups of interest. Based on being above or below the cut-off, participants were assigned to the aforementioned six mutually exclusive groups (see Table 2 for the number of participants in each group).^1^ Third, ANOVA analyses were performed to test for differences between the six groups in baseline levels of CP and psychopathic traits with Bonferroni correction or with Games Howell correction in case the homogeneity of variance assumption was violated. Fourth, five dummy coded group variables (*CP Only*, *Callous-Unemotional Only*, *Psychopathic Personality Only*, *Callous-Unemotional + CP; and Psychopathic Personality + CP*), Parental SES, and gender were *simultaneously* entered as predictors of future CP, aggression, and substance use at 1-, 2- and 3-year follow-up assessments (linear regression analyses) and as predictor of stable CP, aggression and substance use (logistic regression analyses). Statistical analyses were performed by means of SPSS 23.0, and we used *p* < .05 as the indicator of statistical significance if not otherwise specified.

## Results

### Descriptive Information

Table 1 presents correlations between all study variables and shows that the three psychopathic traits dimensions correlated to all antisocial outcomes at each follow-up assessment, both among boys and girls with one exception, being that these traits dimensions were not significantly related to substance use, especially stable substance use in girls. Though not presented in Table 1, significant gender differences (boys > girls) were revealed for all but two variables, being Parental SES and stable aggression (details available upon request).

**Table 1:** Means (SD) and associations between study variables (*N* = 479 boys/517 girls)

	1.	2.	3.	4.	5.	6.	7.	8.	9.	10.	11.	12.	13.	14.	15.	16.	17.	Boys M (SD)	Girls M(SD)
1. Parental SES at baseline^a^	–	−.01 ^ns^	−.08 ^ns^	−.02 ^ns^	−.13**	−.10*	−.13**	−.09 ^ns^	−.06 ^ns^	−.07 ^ns^	−.08 ^ns^	−.06 ^ns^	−.02 ^ns^	.03 ^ns^	−.04 ^ns^	−.04 ^ns^	−.05 ^ns^	4.09 (1.25)	4.02 (1.31)
2. Grandiosity at baseline	−.02 ^ns^	–	.35	.64	.43	.34	.36	.25	.28	.34	.34	.24	.24	.18	.18	.15**	.22	5.63 (3.18)	4.23 (2.74)
3. CU at baseline	−.13**	.31	–	.35	.42	.37	.24	.20	.26	.24	.13**	.11*	.13**	.14**	.11*	.10*	.17	22.77 (8.54)	19.12 (7.86)
4. Impulsivity at baseline	.00 ^ns^	.54	.32	–	.45	.40	.35	.30	.30	.43	.36	.31	.33	.17	.15**	.15**	.18	4.91 (2.56)	4.01 (2.22)
5. CP at baseline	−.11*	.42	.33	.51	–	.54	.47	.39	.47	.45	.42	.35	.38	.19	.25	.23	.27	4.78 (4.14)	2.69 (2.85)
6. CP 1 year follow up	−.13**	.31	.28	.43	.51	–	.61	.51	.66	.83	.52	.44	.50	.42	.33	.34	.35	5.76 (4.84)	3.23 (3.64)
7. CP 2 year follow up	−.12**	.29	.23	.39	.50	.65	–	.70	.64	.52	.85	.61	.56	.24	.54	.43	.37	6.09 (5.37)	3.00 (3.35)
8. CP 3 year follow up	−.12**	.25	.20	.32	.46	.59	.79	–	.65	.44	.61	.83	.54	.18	.41	.57	.35	6.78 (5.36)	3.53 (3.82)
9. Stable CP [n(%)]	−.13**	.19	.09*	.28	.37	.57	.54	.56	–	.55	.54	.55	.62	.24	.36	.42	.39	91 (19%)	27 (5.2%)
10. Aggression 1 year follow up	−.12**	.36	.25	.50	.48	.81	.60	.56	.48	–	.57**	.51	.57	.32	.26	.29	.31	8.72 (5.51)	7.86 (5.12)
11. Aggression 2 year follow up	−.07 ^ns^	.34	.23	.48	.48	.56	.81	.68	.40	.68	–	.67	.60	.18	.45	.34	.30	9.06 (5.96)	8.03 (5.20)
12. Aggression 3 year follow up	−.12**	.32	.18	.38	.45	.52	.70	.82	.42	.62	.77	–	.61	.15**	.32	.47	.33	9.03 (5.98)	8.14 (5.38)
13. Stable aggression [n(%)]	−.09*	.29	.15	.32	.39	.51	.55	.50	.56	.59	.58	.54	–	.18	.20	.32	.34	71 (14.8%)	57 (11.0%)
14. Substance use 1 year follow up	−.09 ^ns^	.11*	.04 ^ns^	.20	.23	.43	.30	.29	.20	.30	.25	.21	.25	–	.27	.24	.32	1.60 (2.65)	0.49 (0.92)
15. Substance use 2 year follow up	−.07 ^ns^	.20	.18	.27	.35	.45	.48	.43	.27	.39	.43	.36	.32	.38	–	.42	.38	1.43 (2.27)	0.46 (0.92)
16. Substance use 3 year follow up	−.16	.13**	.09*	.21	.30	.43	.45	.56	.33	.36	.36	.41	.29	.39	.51	–	.46	1.67 (2.53)	0.61 (1.30)
17 Stable substance use [n(%)]	−.06 ^ns^	.03 ^ns^	.05 ^ns^	.08 ^ns^	.15**	.32	.20	.25	.18	.21	.15**	.15**	.18	.47	.42	.52	–	29 (6.1%)	4 (0.8%)

Values above and below the diagonal are for boys and girls respectively; all correlation coefficients were significant at *p* < .001 unless otherwise specified

*CU*, callous-unemotional traits; *CP*, Conduct Problems

^a^ The educational level of the mother and father were seperately assessed and for each parent ranged from 1 (lowest level) to 6 (highest level). In this table Parental SES reflects the mean score of the sum of two variables, being educational level of the mother and educational level of the father

^ns^ *p* > .05; * *p* < .05; ** *p* < .01

### Baseline Differences Between the Groups

Table 2 shows that *Psychopathic Personality + CP* and *Callous-Unemotional + CP* participants were not significantly different in terms of CP, whereas participants in the *Psychopathic Personality + CP* group were significantly higher in CP than their counterparts in the *CP Only* group. Table 2 also shows that participants in the *Psychopathic Personality Only* group were significantly higher in CU traits than participants in the *Callous-Unemotional Only* group, whereas no significant difference in level of CU traits was revealed between participants in the *Psychopathic Personality + CP* and *Callous-Unemotional + CP* groups. In terms of Grandiosity and Impulsivity, the *Callous-Unemotional Only* and *Callous-Unemotional + CP* groups were merely significantly different in their level of Grandiosity, whereas the *Psychopathic Personality Only* and *Psychopathic Personality + CP* groups were only significantly different in their level of Impulsivity. Table 2 finally shows that a significant higher percentage of girls (versus boys) was assigned to the *Control* group than to most of the other groups. Likewise, the percentage of girls in the *Callous-Unemotional Only* group was significantly higher than in the *Psychopathic Personality + CP* group.

**Table 2:** Group comparisons of baseline levels of conduct problems and psychopathic traits (*n* = 660)^a^

	Control (1) (*n* = 424)	CP only (2) (*n =* 35)	CU only (3) (*n =* 90)	PP only (4) (*n =* 36)	CU + CP (5) (*n =* 14)	PP + CP (6) (*n =* 61)	Group comparisons
	M(SD)	M(SD)	M(SD)	M(SD)	M(SD)	M(SD)	
Conduct Problems*	1.65(1.51)	7.56(1.86)	2.35(1.62)	3.17(1.76)	8.49(3.90)	11.16(3.95)	1 < 2,3,4,5,6; 2,3,4 < 6; 3,4 < 5; 2 > 3,4
Grandiosity*	2.93(1.65)	3.73(1.83)	3.11(1.91)	8.93(1.91)	4.19(1.06)	9.90(1.80)	1 < 4,5,6; 2 < 4,6; 3 < 4,5,6; 4 < 5; 5 < 6; 2 > 3
CU traits*	15.75(5.28)	18.40(4.99)	29.97(3.85)	32.36(4.99)	30.28(5.02)	33.60(7.30)	1 < 2,3,4,5,6; 2 < 3,4,5,6; 3 < 6
Impulsivity	2.78(1.46)	3.57(1.30)	2.78(1.58)	7.16(1.21)	2.82(1.22)	8.09(1.26)	1 < 2,4,6; 2 < 3, 4, 6; 3 < 4, 6; 4 < 5, 6; 5 < 6
Girls [n(%)]	278 (65.6)	10 (28.6)	50 (55.6)	13 (36.1)	3 (21.4)	14 (23.0)	2,4,5,6 < 1; 6 < 3

*CP*, Conduct Problems; *CU*, Callous-Unemotional; *PP*, Psychopathic Personality

* Group comparisons are based on Games Howell post-hoc tests. All differences sig on at least .05 level

^a^N is lower than the total sample (n = 996) because children who were not assigned to one of these six groups were not included in the ANOVAs

### Predicting Future and Stable Conduct Problems, Aggression, and Substance Use

#### Conduct Problems

Table 3 shows that *Psychopathic Personality + CP* was the strongest predictor for CP at each follow-up assessment, followed by *CP Only* and *Psychopathic Personality Only*. *Callous-Unemotional Only* and *Callous-Unemotional + CP* were also prospectively related with future CP, though very weakly and not at all follow-ups. *Psychopathic Personality + CP* was the only significant predictor for stable CP.

**Table 3:** Predicting future and stable conduct problems, future and stable aggression, and future and stable substance use after controlling for parental SES^a^ and gender (*n* = 996)

	Conduct Problems				Aggression				Substance use			
	1 year	2 years	3 years	Stable	1 year	2 years	3 years	Stable	1 year	2 years	3 years	Stable
	β	β	β	OR^a^	β	β	β	OR^a^	β	β	β	OR^a^
CP Only	.06*	.07*	.10**	2.19	.06	.09**	.12***	2.74*	.03	.10**	.07*	3.04
Callous-Unemotional Only	−.05	−.07*	−.05	.33	−.09**	−.10**	−.07*	.18*	−.05	−.03	−.04	.53
Psychopathic Personality Only	.08**	.08**	.10***	2.22	.10**	.09**	.09**	2.89**	.03	.05	.08*	3.79*
Callous-Unemotional + CP	.09**	.06	.06*	2.42	.06	.04	.04	1.52	.04	.06	.03	–
Psychopathic Personality + CP	.32***	.28***	.24***	7.12***	.25***	.25***	.17***	5.20***	.17***	.19***	.15***	7.32***

*β*, Standardized Regression Coefficient; *OR*, Odds Ratio; *CP*, Conduct Problems; *1 year*, 1 year follow-up; *2 years*, 2 year follow-up; *3 years*, 3 years follow-up. Stable, High levels at all three follow-ups

Confidence intervals for unstandardized betas and *OR* are presented in the Supplementary Material

^a^The educational level of the mother and father were separately assessed and for each parent ranged from 1 (lowest level) to 6 (highest level). In all these analyses Parental SES reflects the mean score of the sum of two variables, being educational level of the mother and educational level of the father

*p < .05 **p < .01 ***p < .001

#### Aggression

Table 3 also shows that *Psychopathic Personality + CP* was the strongest predictor for future aggression (at all three follow-ups) and stable aggression, followed by *CP Only*, *Psychopathic Personality*, and *Callous-Unemotional Only*. *Callous-Unemotional + CP* neither was a significant predictor for future aggression nor for stable aggression.

#### Substance Use

Table 3 finally shows that *Psychopathic Personality + CP* was the strongest and most consistent predictor for future and stable substance use. *CP Only* was significantly related with substance use (follow-ups at 2 and 3 years), whereas *Psychopathic Personality Only* was significantly related with future substance use (follow-up at 3 years) and stable substance use. Neither *Callous-Unemotional Only* nor *Callous-Unemotional + CP* were significant predictors for future and stable substance use.

## Discussion

The main aim of the present study was to compare CU- and psychopathy-based subtyping approaches in their ability to predict future and stable forms of various antisocial behaviors. The most robust and strongest prospective relation with future and stable antisocial outcomes occurred when the combination of high levels of interpersonal (i.e., Grandiosity), affective (i.e., CU traits), and behavioral/lifestyle (i.e., Impulsivity) traits co-occurred with high levels of CP at baseline (*Psychopathic Personality + CP*). This main finding and its consistency with prior work suggests that CU traits-based approaches for subtyping children and adolescents with CP are not as efficient as subtyping approaches that use the multidimensional youth psychopathy construct for predicting future and stable antisocial behaviors. The second main finding is that in adolescents *without* CP*,* being high on all three psychopathic traits dimensions (*Psychopathic Personality Only*) often was positively related to future and stable antisocial outcomes, whereas being high on CU traits only (*Callous-Unemotional Only*) was not. Thus, even in the absence of baseline CP, being high on the three psychopathic traits dimensions is a better predictor of future and stable antisocial outcomes than being high on CU traits only.

Following prior work (e.g., Fanti 2013), adolescents could be assigned to *CP Only* and *Callous-Unemotional + CP* groups. Importantly, a substantial number of adolescents with CP was also assigned to the *Psychopathic Personality + CP* group providing evidence for additional heterogeneity in CP. Comparing these three CP groups in terms of baseline levels of CP shows that – as hypothesized – *Psychopathic Personality + CP* youth were higher in baseline CP than the other two CP groups, though it must be noted that the *Psychopathic Personality + CP* and the *Callous-Unemotional + CP Only* groups were not significantly different in baseline CP after correcting for multiple group comparisons. Of note, only a small number of adolescents with CP were assigned to the *Callous-Unemotional + CP* group (*n* = 14), whereas 61 adolescents with CP could be assigned to the *Psychopathic Personality + CP* group. This finding supports the view that being high on CU traits and being high on all three psychopathy dimensions identifies largely overlapping groups of children with CP (Frick 2009), though it is important to note that *Callous-Unemotional + CP* group assignment was not equally predictive as *Psychopathic Personality + CP* group assignment. To illuminate how robust this overlap is, much more work is needed, especially since available evidence on this overlap is mixed (e.g., Colins et al 2018; Christian et al. 1997). Unfortunately, we were not able to use more stringent cut-offs (e.g., > 1.00 SD) to assign the participants to mutually exclusive groups due to sample size restrictions. This limitation may explain why the percentage (55%) of adolescents with CP who were assigned to the* Psychopathic Personality* + *CP* group is too high in light of evidence that the estimated prevalence of Psychopathy Checklist-Revised defined psychopathy in samples of antisocial adults is 15–25% (Drislane and Patrick 2013). As such, future endeavors to find the best way of identifying a relatively small subgroup of CP youth who most strongly and consistently display the features associated with adult psychopathy should be encouraged.

CU traits are considered important for identifying a subgroup of children and adolescents with CP who are at higher risk for future and stable antisocial behaviors (e.g., Frick et al. 2014). Yet, in the present study, *Callous-Unemotional + CP* adolescents were only at risk for future CP at two out of three follow-up assessments, whereas *CP Only* adolescents were at risk for future CP, future and stable aggression, *and* future substance use. *Psychopathic Personality + CP* adolescents were consistently at high risk for all outcomes under consideration at each follow-up assessment, and to a much higher extent than adolescents in any other group, including the *Callous-Unemotional + CP* group. We see at least three potential explanations for this finding. First, impulsivity is a well-documented predictor of various negative outcomes (e.g., Caspi et al. 1996) and mounting evidence shows that interpersonal psychopathic traits are uniquely related to bullying, delinquency, and aggression, sometimes even stronger than CU traits (e.g., Lau and Marsee 2013; Stellwagen and Kerig 2013; Colins 2016). As such, the difference in predictive ability between the two groups may be explained by the higher baseline levels of Grandiosity and Impulsivity seen in the *Psychopathic Personality + CP* group. Second, various studies on the development of antisocial behavior have documented that the higher the number of risk factors, the higher the risk for antisocial behavior (e.g., Stouthamer-Loeber et al. 2002). Thus, the *Psychopathic Personality + CP* (versus *Callous-Unemotional + CP*) group may be at a higher risk for antisocial behavior simply because they display more co-occurring risk factors. Third, it may be, that it is something in the specific combination of the three psychopathic traits dimensions that put youth at a higher risk for negative outcomes. In support for this notion, several studies have found a three-way-interaction effect between the three psychopathic traits dimensions in relation to concurrent and future antisocial behavior in youth (e.g., Colins et al. 2014; Fanti et al. in press; Orue and Andershed 2015).

This study also provides information about the prognostic usefulness of psychopathic traits in youth *without* CP at baseline (i.e., the *Callous-Unemotional Only* and *Psychopathic Personality Only* groups). First, CU traits that occur in the absence of CP or other forms of antisocial behavior are considered to bear clinical significance, for example because *Callous-Unemotional Only* individuals may be at risk for later antisocial behavior (Viding and McCrory 2012). Using the cut-offs of the present study, we do find a rather large group of youths with CU traits without high levels of CP (*n* = 90). The numbers will of course vary with cut-off, but research using other cut-offs and other ways to identify groups, have also found a rather large group of CU only youths (e.g., Fanti 2013). An important question is though whether different cut-offs will yield differences in predictive power. Recent studies testing various cut-offs indicates that different cut-offs does not seem to affect the predictive power (Frogner et al. 2016; Colins et al. 2018; Frogner, Andershed, & Andershed in press). The present study, and these other recent studies (Frogner et al. 2016; Colins et al. 2018; Frogner et al. in press) could not support the notion that CU traits only (i.e., without concurrent CP) may be associated with a high risk for future antisocial behavior, neither when predicting future and stable CP, nor when predicting future and stable aggression and substance use. In fact, *Callous-Unemotional Only* youth were significantly less likely to display future and stable antisocial outcomes, agreeing with prior work (Fanti 2013).

Our findings may also be informative when debating the role of the antisocial dimension (Skeem et al. 2011). Specifically, participants in the present study who were assigned to a psychopathic personality group were at a higher risk for future and stable antisocial behavior when they had high baseline levels of CP (*Psychopathic Personality + CP*) as compared to when they did not (*Psychopathic Personality Only*). This finding dovetails well with evidence showing that adult psychopathy is less predictive of future antisocial behaviors when the antisocial dimension is excluded from the total psychopathy score (e.g., Vitacco et al. 2005). Importantly, our study also suggest that even without introducing a prognostic tautology (in our case CP), being high on all three psychopathic traits dimension helps to identify school-attending adolescents at risk for future CP and other forms of antisocial behavior.

The strengths of the present study include the longitudinal design (3-year follow-up), the use of various negative outcomes (Salekin 2008), and the reliance on measures (APSD and ICU) that have been extensively used in prior work on CU traits and youth psychopathy. Our findings however must be interpreted in the context of several limitations. First, when using higher cut-offs such as .75 *SD* and 1.00 *SD* above the mean as cut-offs, this resulted in groups with too few participants to directly compare the groups of interest with each other. Prior work however, as mentioned previously, shows that the pattern of findings remains substantially similar when using more stringent cut-offs (Colins et al. 2018; Frogner et al. 2016). Nevertheless, to ascertain that our findings can be generalized when using more stringent cut-off scores that are more likely reflective of severe baseline levels of CP and psychopathic traits, studies with larger sample sizes are needed, preferably among clinic-referred and criminal justice-involved individuals. Researchers who already have large data sets available can start to address this issue. Second, our analytical approach (categorizing continuous variables) was based on arbitrary cut-offs and may have resulted in loss of statistical power and increased probability of committing type-I errors (MacCallum et al. 2002). We acknowledge these arguments but also note that despite this assumed reduction in terms of statistical power, we nevertheless found prospective relations between group-membership categories and future and stable antisocial outcomes. Both continuous and categorical approaches are useful and necessary (Lilienfeld 2014), and we encourage researchers to come up with alternatives and better strategies to compare the CU traits only versus multidimensional psychopathy approaches. Third, the present study could only rely on self-report information, and future research that includes various informants will help to overcome the possibility that our prospective relations are largely explained by shared method variance. Fourth, the present study could not specifically test the questions related to *why* there were differences between the various groupings in the prediction of antisocial behavior. A psychopathy based theoretical hypothesis here would be that the *Psychopathic Personality* + CP group is worse off because they exhibit multiple and interacting problematic traits and behaviors, as compared to the other groups studied. Some may however question this and propose that it is merely the higher levels of CU traits in the *Psychopathic Personality* + CP group that explain that groups´ higher risk for future antisocial behavior. These various views could not be tested with the analytical approach used in the present study. Finally, our study did not test the stability of the group assignments and their relation to various antisocial outcomes, and we acknowledge this as a limitation that should be addressed in future research. Filling this void will be important, for example, to learn if youth in the *Psychopathic Personality + CP* group are at a much greater risk to be identified as an adult with psychopathy than their *Callous-Unemotional + CP* counterparts, and to see if youth in the *Callous-Unemotional Only* and *Psychopathic Personality Only* manage to stay under the radar of law enforcement agencies.

The findings of the present study suggest that the entire psychopathy construct outperforms the CU traits alone model in identifying CP youths who are at risk for severe and stable antisocial behavior. These findings together with the fact that psychopathy commonly is defined as a multidimensional construct (e.g., Cooke and Michie 2001; Frick et al. 2000) and often seen as an important risk factor for future antisocial behavior (e.g., DeLisi 2017; Hare 2016) lend support for the notion that researchers may need to consider focusing on the broader psychopathy construct.

If replicated, the present study provides important practical and theoretical information. The findings indicate that the three factor model of psychopathy may be a good definition of psychopathy among youths, since it predicts stable forms of aggression and substance use, albeit not conduct problems. The present study would also indicate that the four factor model of psychopathy (i.e., including concurrent antisocial behavior as a fourth factor) may be a good definition of psychopathy among youths, since this by far was the best predictor of all stable forms of antisocial behaviors in the present study.

Furthermore, in terms of implications for diagnostics and more specifically the Conduct Disorder diagnosis, the present study shows the need for subtyping youths with conduct problems/Conduct Disorder because subgroups with conduct problems (especially the subgroup with concurrent psychopathic personality), show a different level of risk for future antisocial behavior. Youths with conduct problems only (i.e., without concurrent psychopathic traits) showed lower risk for antisocial behavior than the group of youths with conduct problems and concurrent psychopathic personality. In the most recent version of the DSM, only CU traits are used as a specifier for conduct disorder (APA 2013). The current study showed that youth in the *Callous-Unemotional + CP* group were at an increased risk for future CP, though not as much as youth in the *Psychopathic Personality + CP* group. Crucially, *CU + CP* youth were not at an increased risk for future and stable aggression and substance use whereas their *Psychopathic Personality + CP* and even their *CP Only* counterparts were. Further research on the topic will inform future revisions of DSM-5 and ICD-11 if interpersonal and behavioral/lifestyle traits need to be included as additional specifiers for identifying children and adolescents with a psychopathic personality. Being able to differentiate between *CU + CP* and *Psychopathic Personality + CP* youth is not only relevant to avoid that youth who merely display CU traits are misclassified with the stigmatizing label “psychopathic personality’, but it also may help clinicians to identify youth who are at greatest need of intensive treatment due to their chronic and severe engagement in antisocial behavior.

## Electronic supplementary material

ESM 1 (DOCX 14.9 kb)

## Footnotes

1 Group assignments were first performed separately for boys and girls. Unfortunately, this strategy resulted in groups that were too small in number (details available upon request from H.A.). Because gender is an important variable to consider when studying CP and psychopathic traits (e.g., Colins et al. 2017; see also Table 1) gender was entered as a control variable in the linear and logistic regression analyses (see below).
