# Supplementary material for: Callous-Unemotional Traits Only Versus the Multidimensional Psychopathy Construct as Predictors of Various Antisocial Outcomes During Early Adolescence
Source: J Psychopathol Behav Assess. 2018 Mar 9;40(1):16–25. doi: 10.1007/s10862-018-9659-5 (PMC5860126; doi:10.1007/s10862-018-9659-5)
Supplement: Supplementary file 1 — (DOCX 14.9 kb) [file 10862_2018_9659_MOESM1_ESM.docx]

| *Supplementary Table*  Predicting Future and Stable Conduct Problems, Future and Stable Aggression, and Future and Stable Substance Use After Controlling For Parental SES^a^ and Gender (n = 996) with Unstandardized Regression Coefficients and 95% Confidence Intervals, and Odds Ratios (OR) With 95% Confidence Intervals* | | | | | | | | | | | | | | |
| --- | --- | --- | --- | --- | --- | --- | --- | --- | --- | --- | --- | --- | --- | --- |
|  | Conduct Problems | | | |  | Aggression | | | |  | | Substance Use | | |
|  | 1 year | 2 years | 3 years | Stable |  | 1 year | 2 years | 3 years | Stable |  | 1 year | 2 years | 3 years | Stable |
|  | β (CI) | β (CI) | β | OR |  | β | β | β | OR |  | β | β | β | OR |
| CP Only | 1.41  (.02;2.80) | 1.80 (.35;3.25) | 2.61  (1.03;4.19) | 2.19  (.92;5.19) |  | 1.72  (-.05;3.48) | 2.86  (1.02;4.71) | 3.55  (1.65;5.45) | 2.74  (1.22;6.15) |  | .29  (-.40;.99) | .95  (.37;1.53) | .83  (.15;1.52) | 3.04  (.81;11.42) |
| Callous-Unemotional Only | -.70  (-1.59;.19) | -1.12  (-2.15;-.29) | -.83  (-1.84;.19) | .33  (.10;1.08) |  | -1.70  (-2.83;-.57) | -1.91  (-3.10;-.73) | -1.43  (-2.64;-.20) | .18  (.04;.75) |  | -.38  (-.83;.06) | -.20  (-.57;.17) | -.31  (-.75;.13) | .53  (.07;4.08) |
| Psychopathic Personality Only | 1.82  (.46;3.18) | 2.09  (.66;3.51) | 2.80  (1.25;4.36) | 2.22  (.91;5.43) |  | 2.78  (1.04;4.52) | 2.68  (.86;4.49) | 2.79  (.91;4.66) | 2.89  (1.33;6.43) |  | .37  (-.31;1.06) | .46  (-.11;1.04) | .86  (.19;1.53) | 3.7  (1.02;14.12) |
| Callous-Unemotional + CP | 3.50  (1.21;5.79) | 2.37  (-.03;4.77) | 2.62  (.01;5.24) | 2.42  (.62;9.42) |  | 2.78  (-.14;5.70) | 1.83  (-1.22;4.87) | 2.25  (-.90;5.40) | 1.52  (.32;7.12) |  | .70  (-.45;1.85) | .96  (-.001;1.92) | .55  (-.58;1.68) | .00  (na) |
| Psychopathic Personality + CP | 5.97  (4.91;7.04) | 5.39  (4.27;6.51) | 5.01  (3.80;6.23) | 7.12  (3.96;12.80) |  | 5.47  (4.11;6.83) | 5.74  (4.33;7.16) | 3.92  (2.45;5.38) | 5.20  (2.91;9.29) |  | 1.44  (.91;1.98) | 1.42  (.97;1.86) | 1.30  (.77;1.83) | 7.32  (3.12;17.17) |
| *Note*. β = Unstandardized Regression Coefficient; OR = Odds Ratio*;* CP = Conduct Problems; 1 year = 1 year follow-up; 2 years = 2 year follow-up; 3 years = 3 years follow-up; Stable, High levels at all three follow-ups.  a The educational level of the mother and father were separately assessed and for each parent ranged from 1 (lowest level) to 6 (highest level). In all these analyses, Parental SES reflects the mean score of the sum of two variables, being educational level of the mother and educational level of the father  * Only 95% CIs are presented. Other Cis (99% and 99.99%) are available upon request | | | | | | | | | | | | | | |
